# Vitamin D Deficiency is Associated With Dengue Severity in Pediatric Cases, Eastern India

**DOI:** 10.1155/jotm/2863024

**Published:** 2025-04-03

**Authors:** Sagnika Samal, Manoj Kumar Dash, Chinmay Kumar Behera, Gyanraj Singh, Mahesh Chandra Sahu, Biswadeep Das

**Affiliations:** ^1^School of Biotechnology, KIIT-Deemed to be University, Bhubaneswar 751024, Odisha, India; ^2^Department of Pediatrics, Kalinga Institute of Medical Sciences, KIIT-Deemed to Be University, Bhubaneswar 751024, Odisha, India; ^3^Department of Anatomy, Jajati Keshari Medical College and Hospital, Jajpur 755001, Odisha, India; ^4^ICMR-Regional Medical Research Centre, Bhubaneswar 751023, Odisha, India

**Keywords:** 25(OH)D deficiency, dengue fever, dengue virus, dengue with warning signs, severe dengue

## Abstract

Vitamin D is an important modulator of host immune response during immunity development in context to several diseases in children. This study included 280 pediatric dengue cases, which were further stratified as dengue fever (DF), dengue with warning signs (DWS), and severe dengue (SD), wherein naturally circulating vitamin D metabolite, 25(OH)D was assessed, followed by statistical analysis for elucidating the role of serum 25(OH)D in dengue severity. qRT-PCR based serotyping showed that dengue virus (DENV)-2 was the major circulating DENV serotype in the patients. Among DENV cases, 92 (32.86%), 108 (38.57%), and 80 (28.57%) were diagnosed with DF, DWS, and SD, respectively. Among SD patients, serum 25(OH)D deficiency and insufficiency were observed in 37 (46.25%) and 43 (53.75%) patients, respectively, and found to be significantly associated with SD (*p* < 0.05) in comparison to DF and DWS. Serum 25(OH)D sufficiency had a protective effect against dengue severity and could be a determinant for DENV outcome in children.

## 1. Introduction

Dengue virus (DENV) infection is one of the most important public health related arthropod-borne viral disease [1]. DENV is estimated to affect 100–400 million people annually in more than 100 countries [2]; India being a leading contributor for DENV infections, wherein Odisha State of India has been consistently reporting substantial number of dengue cases each year [3–5]. DENV infection can manifests as dengue fever (DF), which is mild and normal fever with rash, headache, muscle ache, joint pain, and nausea; dengue with warning signs (DWS) that includes all components of DF in addition to petechia, mucosal bleeding, persistent vomiting, abdominal pain or tenderness, increased hematocrit, with thrombocytopenia; and severe dengue (SD), which includes all components of DWS in addition to vascular permeability, significant increase in hematocrit leading to plasma leakage with hypovolemia and shock, thrombocytopenia, severe bleeding, organ impairment and coagulopathy [6]. Initial manifestation of DENV is DF; however, as the infection progresses, the individual may develop DWS or SD depending on a variety of factors, including immune status, infected serotype, viral load, age, and nutrition [7], of which host immunity is a crucial factor [1]. Hence, modulators of host immune response could play a crucial role in delineating the progression of DF to SD in patients. Vitamin D is considered an important immune modulator and has been reported to modulate the innate immunity in infectious and noninfectious diseases, because it has strong immunomodulatory activity and antiproliferative responses [8, 9]. Studies in the Indian subcontinent populations have shown the association of vitamin D levels with dengue, wherein some studies reported high serum vitamin D levels in SD cases [10], and few studies depicted low serum vitamin D levels in SD cases [2, 11–15]. Considering the prevalence of SD in children [4], and the fact that children possess a developing immune system, it is important to assess the vitamin D status in the pediatric dengue patients for evaluating the association between vitamin D and dengue severity.

## 2. Materials and Methods

**Figure 1:**
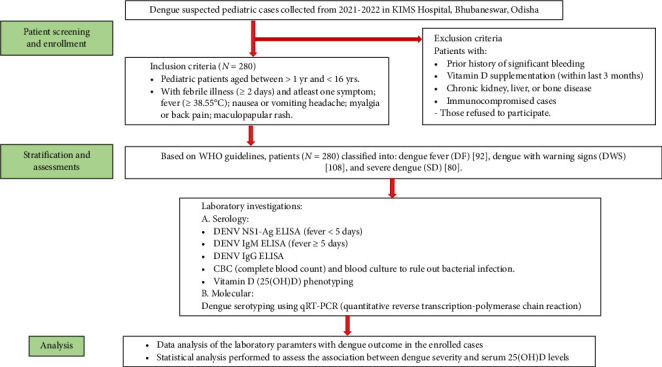
Flowchart representing subject enrollment process during the study period (2021-2022).

This study assessed the vitamin D status in pediatric dengue patients that were sampled by the clinicians of Pediatrics Department, Kalinga Institute of Medical Sciences (KIMS), Bhubaneswar, Odisha, India over a period of two years (2021-2022). The study was approved by the Institute Ethical Clearance of KIMS, Bhubaneswar (Ref No. KIIT/KIMS/IEC/1094/2022). Inclusion criteria were all children (> 1 year and < 16 years) with two or more days of febrile illness along with one or more of the following symptoms: fever ≥ 38.5°C, nausea or vomiting, headache, myalgia, back pain or a maculopapular rash with no other signs of obvious infection elsewhere. Blood was collected from the patients on the first day of hospital admission, after evaluation of clinical manifestations of DF. Exclusion criteria were patients with significant bleeding prior to study enrolment, those taking vitamin D supplements in last 3 months, and those with chronic diseases of kidney, liver, and bone, immunocompromised patients, and patients unwilling to participate in the study. Patients with a history of previous DENV infection (within a year) were excluded from the analysis so as to rule out the possibility of antibody dependent enhancement (ADE) that could lead to dengue severity. Serological tests, DENV NS1-Ag ELISA, DENV IgM ELISA, and DENV IgG sandwich ELISA was conducted in all the collected samples. In addition, routine biochemical tests, complete blood count (CBC), and blood culture were performed to rule out any evidence of bacterial infection. Of the clinically suspected patients sampled, a total of 280 laboratory confirmed DENV pediatric patients belonging to East India origin were included in the study. The patients positive for one or more serological tests were segregated into three groups: DF, DWS, and SD based on clinical manifestations as per WHO [16]. DENV serotyping was performed using quantitative reverse transcriptase polymerase chain reaction (qRT-PCR) using NS5, E, and M primers in separate reactions with slight modifications [17]. Briefly, viral cDNA was synthesized at 50°C for 30 min and amplified using the following reagents: NS5 and M primers for DENV-1 and DENV-3, and E and E-M primers for DENV-2 and DENV-4 serotyping, which were further mixed with SyBr Green in separate reactions. The reaction conditions utilized for DENV-2 and DENV-4 were: initial denaturation at 95°C for 5 min, followed by 45 cycles of denaturation at 95°C for 15 s, annealing at 58°C for 30 s, and extension at 60°C for 30 s and for DENV-1 and DENV-3, initial denaturation at 95°C for 5 min, followed by 45 cycles of denaturation at 95°C for 15 s and annealing at 60°C for 1 min [17]. Vitamin D status in the subjects was assessed based on measuring the levels of circulating serum 25(OH)D metabolite; sufficient (> 20 ng/mL), insufficient (12–20 ng/mL) and deficient (< 12 ng/mL) [18, 19], using COBAS e 411 immunoassay system (ROCHE Diagnostics GMBH, Sandhofer Stn. 116, D-68305, Manheim, Germany) [18]. The data obtained were analyzed by Chi-square test to compare the differences between the specific characteristics such as age, sex, thrombocytopenia, and hematocrit among the three dengue groups. One way-ANOVA followed by tests of multiple comparison was performed to compare the mean serum 25(OH)D among the three groups. To assess if serum 25(OH)D is associated with dengue severity, a simple logistic regression analysis was performed between sub groups (DF vs. DWS; DWS vs. SD; DF vs. SD), along with estimating the odds ratio for the analysis using R Studio 4.3.1, and a *p* value < 0.05 considered statistically significant for all analyses (Figure 1).

## 3. Results

All the patients were serologically positive for DENV-IgM and/or DENV-NS1 antigen, depicting primary acute DENV infection. Of the laboratory confirmed cases, a total of 246 patients were positive for both DENV-NS1 antigen and DENV-IgM, and 34 patients were positive for DENV-IgM only. No patient was positive for DENV-IgG, excluding the presence of secondary DENV infection. qRT-PCR based serotyping showed that DENV-2 was the major circulating DENV serotype in 248 patients, whereas remaining patients (*n* = 32) were PCR-negative and only DENV-IgM positive (Supporting Figure S1). Serum 25(OH)D in serum samples varied across dengue patients as per clinical severity; 48 (17%), 155 (55.3%), and 77 (28%) patients had serum 25(OH)D sufficiency, insufficiency and deficiency, respectively (Supporting Table S1). Serum 25(OH)D deficiency and insufficiency was observed in 37 (46.25%) and 43 (53.75%) of SD patients, respectively. Thrombocytopenia and high hematocrit were significantly associated with dengue outcomes in context to severity (*p* < 0.05) (Table 1).

**Figure 2:**
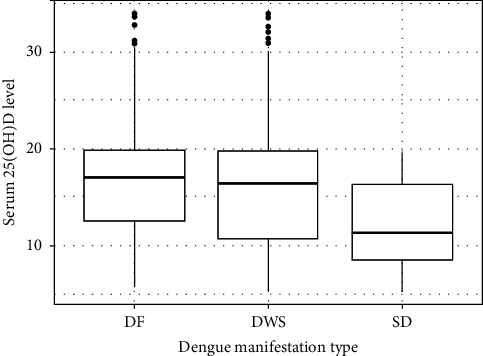
Box plot depicting that median serum 25(OH)D level significantly decreased in SD cases compared to DWS and DF (DF: dengue fever, DWS: dengue with warning signs, SD: severe dengue). SD was found to be significantly associated with vitamin D deficiency and insufficiency among the three groups (asterisks) (*p* < 0.05, ANOVA test).

Serum 25(OH)D sufficiency was not reported in SD cases, although serum 25(OH)D sufficiency was observed in DF and DWS (Figure 2). Simple logistic regression analysis revealed that serum 25(OH)D sufficiency could act as a protective factor against dengue severity in children [OR: 0.86 *p* < 0.0001 for groups (DF vs. SD); OR: 0.85, *p* < 0.0001 for groups (DWS vs. SD), OR: 0.992, *p*=0.423 for groups (DF vs. DWS)] (Supporting Table S2).

## 4. Discussion

The present study depicted that there was a substantial reduction in dengue severity with increasing levels of serum 25(OH)D, and the risk of progression from DF to DWS to SD was significantly reduced. Such an observation could be attributed to the host immunomodulation in context to vitamin D expression during DENV infection. Recent in vitro studies showed that vitamin D influenced the differentiation of monocytes to macrophages by altering the expression of mannose receptor and pro-inflammatory cytokine production in response to DENV infection, and deficiency in vitamin D could elevate immature macrophages [11, 20]. Studies also reported that monocyte-derived dendritic cells (MDDCs) differentiated in a serum-dependent way and underwent a greater resistance to DENV-2 serotype compared to controls, who did not receive high doses of vitamin D [11, 21]. Interestingly, there exists few conflicting evidence regarding the relationship between serum 25(OH)D and DENV infection. Several studies have reported the association of low levels of circulatory 25(OH)D with dengue severity [2, 11–15, 22], whereas few other studies reported that SD patients exhibited higher levels of circulatory 25(OH)D [10], which indicated that the precise underlying mechanism for such modulation of DENV severity with regards to vitamin D status is not clarified to date. Few limitations of the study could be no DENV-1, DENV-3, and DENV-4 serotypes in the study population that could have further corroborated the association of Vitamin D with all DENV serotypes during infection, and no secondary infection patient was obtained in the study that might elucidate the role of vitamin D in secondary infection. Hence, more clinical studies on pediatric DENV infection will provide substantial evidence to elucidate the role of vitamin D in delineating dengue severity.

## 5. Conclusion

In conclusion, the present study depicts the association of serum 25(OH)D deficiency with dengue severity in pediatric cases. Natural serum 25(OH)D sufficiency in children could be protective against dengue severity owing to its immunomodulatory effects in the developing immunity in children, and could alleviate dengue outcome.

## Figures and Tables

**Table 1:** Association of demographic and specific laboratory parameters in pediatric dengue cases (*N* = 280), classified into DF (dengue fever), DWS (dengue with warning signs), and SD (severe dengue).

Characteristics	Number (%)	DF	DWS	SD	*p* value
Age (years)					
2–6	137 (48.9)	45	55	37	NS
7–16	143 (51.1)	47	53	43	
Gender					
Male	141 (50.4)	51	51	39	NS
Female	139 (49.6)	41	57	41	
Thrombocytopenia					
No	133 (47.5)	56	46	31	< 0.05^∗^
Yes	147 (52.5)	36	62	49	
Hematocrit (> 40%)					
No	148 (52.9)	66	46	36	< 0.05^∗^
Yes	132 (47.1)	26	59	47	

Abbreviation: NS = nonsignificant.

^∗^Statistically significant.

## Data Availability

The data that supports the findings of this study are included in manuscript and supporting information of this article.

## Supporting Information

Supporting Information 1 Supporting Figure S1: SYBr green-based qRT-PCR amplification curves indicating detection of DENV-2 in clinical samples with cycle threshold (Ct) values ranging from 16 to 24.

Supporting Information 2 Supporting Table S1: Serum 25-hydroxyvitamin D [25(OH)D] concentrations and their distributions across different clinical manifestations of dengue fever (DF), dengue with or without warning signs (DWS), and severe dengue (SD). The table classifies 25(OH)D level into three categories: < 12 ng/mL (deficiency), 12–20 ng/mL (insufficiency), and > 20 ng/mL (sufficiency) for each clinical group.

Supporting Information 3 Supporting Table S2: Serum 25-hydroxyvitamin D [25(OH)D] levels used in binary logistic regression analysis for different clinical manifestations of dengue. The table represents 25(OH)D levels for patients with dengue fever (DF), dengue with or without warning signs (DWS), and severe dengue (SD), categorized to assess the association of vitamin D levels with disease severity.
