# Supplementary figures and images for: Vitamin D Deficiency is Associated With Dengue Severity in Pediatric Cases, Eastern India
Source: J Trop Med. 2025 Apr 3;2025:2863024. doi: 10.1155/jotm/2863024 (PMC11991798; doi:10.1155/jotm/2863024)

## Slide 1
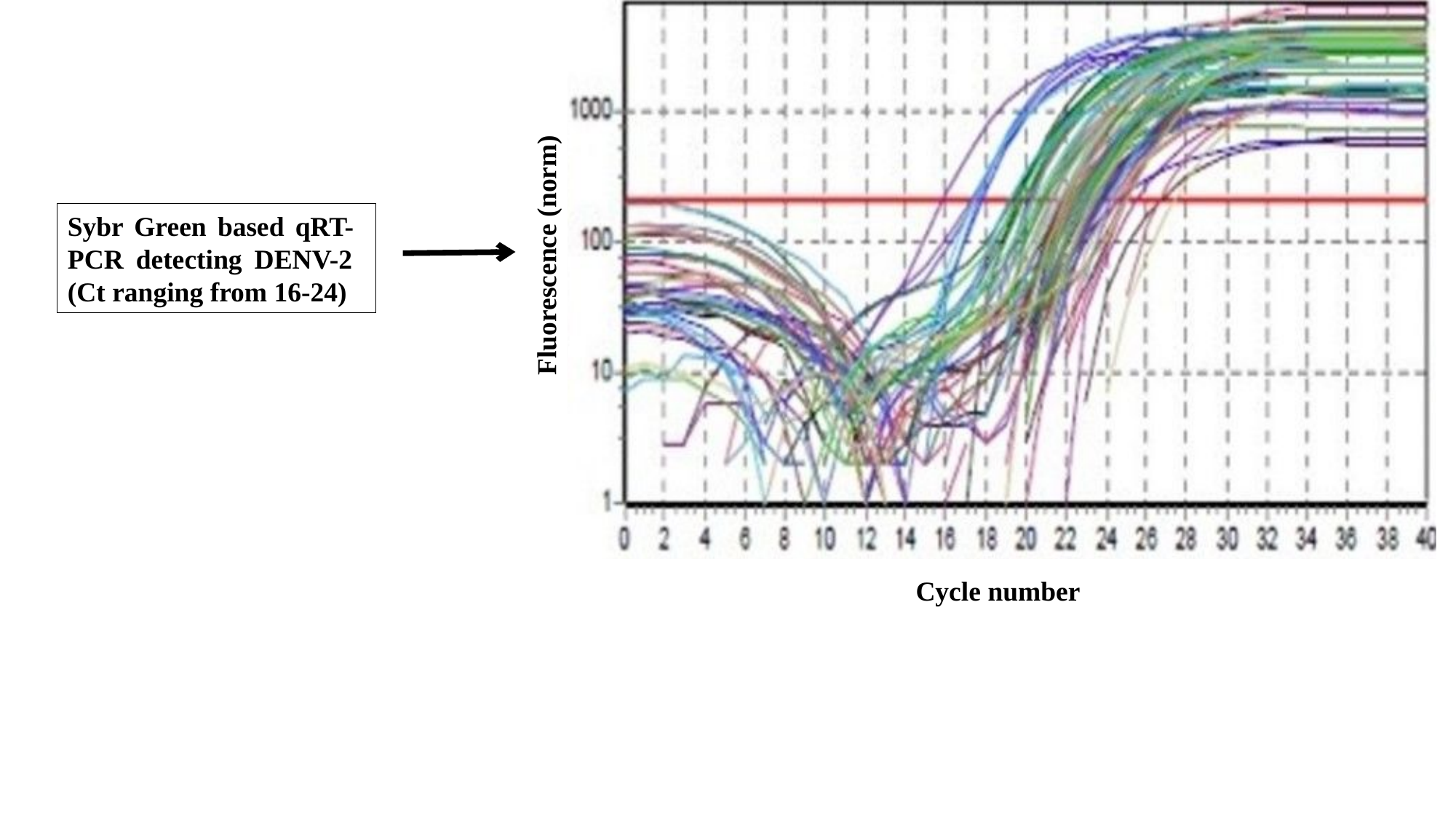

Sybr Green based qRT- PCR detecting DENV-2 (Ct ranging from 16-24)
Fluorescence (norm)
Cycle number

Supplement: Supporting Information 1 — Supporting Figure S1: SYBr green-based qRT-PCR amplification curves indicating detection of DENV-2 in clinical samples with cycle threshold (Ct) values ranging from 16 to 24. [file 2863024.f1.pptx]
